# Identification of prognosis-related lncRNAs and cell validation in lung squamous cell carcinoma based on TCGA data

**DOI:** 10.3389/fonc.2023.1240868

**Published:** 2023-10-25

**Authors:** Yishuang Cui, Yanan Wu, Mengshi Zhang, Yingze Zhu, Xin Su, Wenyue Kong, Xuan Zheng, Guogui Sun

**Affiliations:** ^1^ School of Public Health, North China University of Science and Technology, Department of Hebei Key Laboratory of Medical-Industrial Integration Precision Medicine, North China University of Science and Technology Affiliated Hospital, Tangshan, Hebei, China; ^2^ Department of Hebei Key Laboratory of Medical-Industrial Integration Precision Medicine, North China University of Science and Technology Affiliated Hospital, Tangshan, Hebei, China

**Keywords:** LUSC, lncRNA, LINC00923, LINC01341, TCGA

## Abstract

**Objective:**

To discern long non-coding RNAs (lncRNAs) with prognostic relevance in the context of lung squamous cell carcinoma (LUSC), we intend to predict target genes by leveraging The Cancer Genome Atlas (TCGA) repository. Subsequently, we aim to investigate the proliferative potential of critical lncRNAs within the LUSC milieu.

**Methods:**

DESeq2 was employed to identify differentially expressed genes within the TCGA database. Following this, we utilized both univariate and multivariate Cox regression analyses to identify lncRNAs with prognostic relevance. Noteworthy lncRNAs were selected for validation in cell lines. The intracellular localization of these lncRNAs was ascertained through nucleocytoplasmic isolation experiments. Additionally, the impact of these lncRNAs on cellular proliferation, invasion, and migration capabilities was investigated using an Antisense oligonucleotides (ASO) knockdown system.

**Results:**

Multivariate Cox regression identified a total of 12 candidate genes, consisting of seven downregulated lncRNAs (BRE-AS1, CCL15-CCL14, DNMBP-AS1, LINC00482, LOC100129034, MIR22HG, PRR26) and five upregulated lncRNAs (FAM83A-AS1, LINC00628, LINC00923, LINC01341, LOC100130691). The target genes associated with these lncRNAs exhibit significant enrichment within diverse biological pathways, including metabolic processes, cancer pathways, MAPK signaling, PI3K-Akt signaling, protein binding, cellular components, cellular transformation, and other functional categories. Furthermore, nucleocytoplasmic fractionation experiments demonstrated that LINC00923 and LINC01341 are predominantly localized within the cellular nucleus. Subsequent investigations utilizing CCK-8 assays and colony formation assays revealed that the knockdown of LINC00923 and LINC01341 effectively suppressed the proliferation of H226 and H1703 cells. Additionally, transwell assays showed that knockdown of LINC00923 and LINC01341 significantly attenuated the invasive and migratory capacities of H226 and H1703 cells.

**Conclusion:**

This study has identified 12 candidate lncRNA associated with prognostic implications, among which LINC00923 and LINC01341 exhibit potential as markers for the prediction of LUSC outcomes.

## 1 Introduction

Lung cancer consistently ranks as the leading cause of global cancer-related mortality (1, 2), with lung squamous cell carcinoma (LUSC) exhibiting a heightened propensity for metastasis and recurrence (3–5). In recent years, molecular targeted therapy has substantially enhanced the survival rates of patients afflicted with various malignancies. However, the progress in the realm of molecular targeted therapy for LUSC patients has been notably sluggish (6–8). In order to gain a deeper understanding of the biology of LUSC, the development of robust modeling systems assumes paramount importance (9–11).

Although long non-coding RNAs (lncRNAs) may not possess the same level of evolutionary conservation as protein-coding genes, their promoter regions manifest significant sequence conservation, underscoring the critical nature of lncRNA regulation (12–18). In healthy tissues, lncRNAs are subject to stringent regulation, but in the context of disease, they often succumb to dysregulation, culminating in aberrant expression patterns (19–23). While the mechanistic underpinnings of a subset of lncRNAs have been comprehensively elucidated in the context of LUSC, the vast majority of these molecules remain enigmatic (24–26). Consequently, the present study embarked on the identification of prognosis-related lncRNAs for LUSC, drawing upon the wealth of data within The Cancer Genome Atlas (TCGA) database. Subsequently, we conducted an extensive examination encompassing cell line models, proliferation assays, invasion assessments, and migration studies, all directed toward deciphering the potential of two upregulated lncRNAs in LUSC as predictive biomarkers.

## 2 Materials and methods

### 2.1 Sample source

Two tiers of data, denoted as Level 1 and Level 4, were retrieved from the TCGA repository. Level 4 primarily encompasses fundamental patient attributes, including sex, age, race, and the Tumor Lymph Node Metastasis (TNM) staging; whereas Level 1 provides more detailed information, including specifics such as medication dosage, treatment efficacy, radiotherapy records, and other relevant information for each follow-up day. In total, 504 LUSC tumor samples were procured for analysis. RNAseq expression values, which had been corrected utilizing Recursive Structural Equation Model (RSEM), were obtained from the Level 3 dataset of the LUSC project within the TCGA database hosted at Firehost (https://gdac.broadinstitute.org/). Subsequently, known lncRNA expression values were discerned from this dataset, and a total of 51 normal samples and 501 tumor samples were included in the ensuing analysis.

### 2.2 Differential analysis of lncRNA expression

DESeq2 software was employed to analyze the differential expression of lncRNAs between normal and cancer samples. The lncRNA expression values were subjected to statistical analysis with a predefined significance threshold (*p* < 0.05, |log2FoldChange| > 1). The software was configured accordingly to derive the differentially expressed lncRNA profiles.

### 2.3 Screening for lncRNA related to prognosis

We first compiled a list of lncRNAs associated with survival outcomes through single-factor Cox proportional hazard regression analysis. Subsequently, we conducted multivariable Cox proportional hazard regression analysis to identify independent prognostic factors influencing survival. Finally, a stepwise selection procedure was employed in the context of multivariable Cox proportional hazard regression analysis to identify lncRNAs with significant prognostic relevance based on their associated *P*- values.

### 2.4 Prediction of lncRNA target genes

The identification of potential lncRNA (in cis acting) target genes was performed by screening for differentially expressed genes within 10 kb upstream and downstream of the lncRNAs. This approach is grounded in the recognition that interactions between lncRNAs and their target genes typically occur in close genomic proximity. The selection of a 10-kilobase range serves to focus the research, mitigate computational complexity, and enhance prediction accuracy. In contrast, the identification of potential lncRNA target genes acting in a trans manner commenced with an initial screening using the blast algorithm (e-value < 1e-5) and was subsequently refined using RNAplex software (G<-20). It is worth noting that lncRNAs exert their influence on mRNA expression through various mechanisms, including: 1) direct cis and trans interactions with mRNAs; 2) binding to microRNAs (miRNAs), thereby impeding miRNA-mediated mRNA regulation; and 3) binding to RNA-binding proteins (RBPs), influencing RBP-mRNA interactions. The scope of this analysis was limited to the examination of RNA-seq data from the TCGA database, which exclusively provides expression profiles of lncRNAs and mRNAs. Therefore, the initial phase of analysis focused on discerning potential interactions between these molecules at the expression level through correlation analysis.

### 2.5 Analysis of Kyoto Encyclopedia of Genes and Genomes and Gene Ontology functional enrichment of lncRNA target genes

For KEGG analysis, the p-value was determined utilizing Fisher’s exact test. Signal transduction and disease pathways exhibiting statistical significance with a threshold of *p* < 0.05 were selected for further analysis. For GO analysis, the p-value was computed employing the hypergeometric distribution method. Annotations characterized by a high frequency and possessing a p-value less than 0.05 were retained for subsequent investigation.

### 2.6 RNA extraction and qPCR experiment

RNA extraction was performed employing the Jinbaite RNA Extraction Kit. Subsequently, DEPC water was introduced, and the concentration of RNA was quantified. Quantitative polymerase chain reaction (qPCR) experiments were executed utilizing the Zhongshi Tongchuang Reverse Transcription and Amplification Kit. The primer sequences employed are detailed in **Table 1**.

**Table 1:** Primer sequences.

Primer	Sequences
h-LINC00923_qPCR_97bp_F1	CACTCTCATGGCGTCCTCCT
h-LINC00923_qPCR_97bp_R1	GGTCTTCTCCTTGTCCTCACTCC
h-LINC01341_qPCR_76bp_F1	ACTTTACCGTCGGCATTTGTG
h-LINC01341_qPCR_76bp_R1	TGCTGGGTGTCTTTGACTCTCA
h-CCL15-CCL14_qPCR_156bp_F1	TCGGTCTCTCACTCTGCCTTAT
h-CCL15-CCL14_qPCR_156bp_R1	GAATGCTGCCTTTTTTCCCTT
h-BRE-AS1_qPCR_139bp_F1	CAGCACCTTTGAGCGATGG
h-BRE-AS1_qPCR_139bp_R1	CGAGCCGCAGACTGAGTAACT
h-DNMBP-AS1_qPCR_117bp_F1	TTATGCACTGTGCTAAATCTCAACC
h-DNMBP-AS1_qPCR_117bp_R1	TCAGTTACTCGTGCTTCTCCTCAG
h-LINC00482_qPCR_71bp_F1	CGCACGCTTTAATCAAGGAC
h-LINC00482_qPCR_71bp_R1	CAGCTCACGACACCCATGTAG
h-LOC100129034_qPCR_104bp_F1	AAGAGTGTCATTAGTGAACACGGC
h- LOC100129034_qPCR_104bp_R1	TGTCAAGGGACCAAGTGCTTC
h-MIR22HG_qPCR_120bp_F1	CAAGAACCATCTGCGAAAGGA
h-MIR22HG_qPCR_120bp_R1	TGCTTCCAGCTCTATTTGCCT
h-PRR26_qPCR_96bp_F1	AAATAGCTTGACACCTCCTGCG
h-PRR26_qPCR_96bp_R1	CCCTCCAGTGTTGACTCTGCTG
h-FAM83A-AS1_qPCR_160bp_F1	GCCACTCAGCAATTTTTCTTGA
h-FAM83A-AS1_ qPCR_160bp_R1	TTCTTCTGGTTGTATATGGTTCTCC
h-LINC00628_qPCR_73bp_F1	AACCCACGCCCTCCTGAAT
h-LINC00628_qPCR_73bp_R1	TGCCGCTCCATAAATGCTACT
h-LOC100130691_qPCR_130bp_F1	TGCCTCAGTTATCAACACACACC
h- LOC100130691_qPCR_130bp_R1	TGACCTTTCCACTTAAGCCATC
h-GAPDH_qPCR_309bp_F1	GAACGGGAAGCTCACTGG
h- GAPDH _qPCR_309bp_R1	GCCTGCTTCACCACCTTCT
h-U6_qPCR_251bp_F1	CTCGCTTCGGCAGCACA
h-U6_qPCR_251bp_R1	AACGCTTCACGAATTTGCGT
ASO-h-LINC01341_F1	TCCAGCAGTGGTGCCATGTT
ASO-h-LINC01341_ R1	GATGGCAGCAAGCAAGCTTC
ASO-h-LINC00923_ F1	CCTATGTCCTGTAAAACGCC
ASO-h-LINC00923_ R1	CCCTGCGATGTGGAAAATTC

### 2.7 Nuclear cytoplasmic separation experiment

The isolation of nuclear and cytoplasmic RNA was accomplished employing Norgen’s RNA Nucleocytoplasmic Separation Kit in strict accordance with the manufacturer’s provided instructions. H226 and H1703 cells were collected into enzyme-free EP tubes, and 30 μL of solution buffer was added to each. The procedure began with an initial centrifugation step at 2000 rpm for two minutes, followed by a subsequent centrifugation at 14000 rpm for one minute. Upon removal of the supernatant, lysis buffer was introduced, and centrifugation was repeated. The supernatant, denoted as “tube 2”, was carefully transferred to a new tube, while the original EP tube’s precipitate retained the nuclear RNA and was labeled as “tube 1”. To both tube 1 and tube 2, 400μL of Buffer SK was added, followed by 200 μL of Buffer SK. Vortex mixing for 10 seconds was carried out prior to the addition of 200μL of anhydrous ethanol, followed by further vortex mixing for 10 seconds. The resultant mixture was subsequently loaded into centrifuge columns, with one column allocated for the nuclear fraction and another for the cytoplasmic fraction. Centrifugation was performed at 12000 rpm for one minute, facilitating the separation of nucleic material. Subsequently, the elution process involved the removal of the flow-through liquid from the collection tube, followed by the addition of 400uL of Wash Solution A, accompanied by centrifugation at 12000 rpm for one minute. This washing step was repeated thrice. Finally, a centrifugation at 12000 rpm for two minutes was conducted, after which the centrifuge columns were transferred to new tubes. Here, 50μL of Elution Buffer E was added, and further centrifugation ensued. The resultant eluate was collected, and the RNA concentration was measured.

### 2.8 Cell transfection

Preparation for transfection commenced with the addition of 500μL of Opti-MEM serum-free medium approximately 30 minutes prior to the transfection process. Transfection mixture A, intended for the introduction of one plasmid into each well of a 6-well plate, was created as follows: plasmid DNA (3 µg) was combined with Opti-MEM serum-free medium to achieve a final volume of 50 µL, and the resultant mixture was gently homogenized. Transfection mixture B was prepared by adding 3μL of Lipofectamine 2000 (Invitrogen, USA) to Opti-MEM serum-free medium until the final volume reached 50 µL. This mixture was subsequently thoroughly mixed and allowed to incubate for a duration of five minutes. The two distinct solutions, transfection mixture A and transfection mixture B, were then merged and incubated for an additional 20 minutes prior to their introduction into the target cells. Subsequently, following a 6-hour incubation period, 1.5 mL of complete medium was introduced.

### 2.9 CCK-8 experiment

100 μL of cells at a concentration of 1×10^4^ were dispensed into each well of a 96-well plate, with three replicates per group, ensuring that the periphery of the plate was fully surrounded by sterile phosphate-buffered saline (PBS). Following a 48-hour incubation period, 10 μL of the CCK-8 assay solution were added to each well while taking care to prevent bubble formation. Subsequently, the 96-well plate was incubated at room temperature for one hour before being subjected to machine-based testing.

### 2.10 Colony formation assay

Following a 48-hour period post-transfection, 5×10^3^ cells from each experimental group were inoculated onto individual wells of a 6-well plate, with three wells allocated per sample. Subsequently, crystal violet staining was performed once cellular clusters had formed.

### 2.11 Transwell assay

To conduct the invasion experiment, an extracellular matrix coating solution was meticulously prepared, and precisely 50 µL of this solution was aseptically dispensed into each well. Subsequently, a serum-free cell suspension, comprising 2×10^5^ cells per well, was introduced into the upper compartment of the transwell system. The lower compartment was filled with a culture medium supplemented with 20% Fetal Bovine Serum (FBS). Following a 48-hour incubation period, the transwell chambers were carefully extracted. The cells were subsequently subjected to fixation using methanol for a duration of seven minutes, followed by a 7-minute staining procedure with crystal violet. Following the staining protocol, the cells underwent a thorough washing process. The membrane, bearing the adherent cells, was affixed onto a microscope slide and subsequently subjected to microscopic examination, with three random fields captured for further analysis. The migration experiment, in contrast, did not entail the utilization of matrix adhesive. However, all other procedural steps remain consistent with those employed in the invasion experiment.

### 2.12 Statistical analysis

In this study, statistical analyses were conducted utilizing the Student’s t-test and the χ^2^ test. The difference between two groups was assessed via a two-tailed t-test analysis, with a significance threshold set at *p* < 0.05. Survival analysis was conducted employing the Kaplan−Meier method, and intergroup differences among patients were compared utilizing the log-rank test.

## 3 Results

### 3.1 Sample source and differential lncRNA analysis

**Figure 1:**
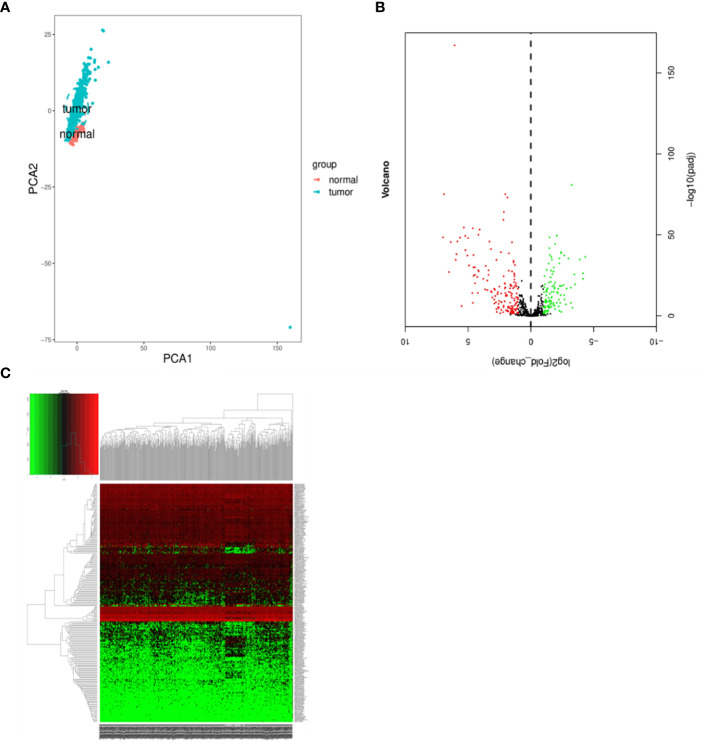
Differentially expressed lncRNAs in samples. **(A)** Principal component analysis. **(B)** Volcano plot visualization of differential lncRNA expression. **(C)** Chart depicting clustering analysis of lncRNAs. In Figure C, the horizontal axis illustrates the clustering of samples, with each column representing an individual sample. The vertical axis denotes the substantial distinctions in lncRNA clustering. The color scale reflects the abundance of lncRNA expression, where red signifies elevated lncRNA expression within the sample, while green signifies lower expression levels. Deeper shades of color indicate more pronounced differences in expression levels.

We conducted a comprehensive statistical analysis of key clinical attributes pertaining to LUSC patients, as delineated in **Table 2**. Principal component analysis (PCA) indicated that the selected samples could be effectively categorized into a single cohesive group for subsequent analyses, as presented in **Figure 1A**. Subsequently, a rigorous differential expression analysis of lncRNAs was undertaken on the samples, yielding a volcano plot that depicted the expression variations across all lncRNAs. Within this analysis, a total of 160 lncRNAs exhibited a significant upregulation, while 110 lncRNAs displayed a significant downregulation, as illustrated in **Figure 1B**. In order to screen for lncRNAs with potential synergistic effects or similar regulation, and to help explore the functions of lncRNAs, clustering analysis was conducted. The clustering analysis results showed the expression trend of all differential lncRNAs in all samples, as shown in **Figure 1C**.

**Table 2:** Clinical attributes of LUSC patients.

clinical attributes	category	number	percentage (%)
Age	<60	91	18.06
	≥60	403	79.96
	NA	10	1.98
	Total	504	100
Sex	Female	131	25.99
	male	373	74.01
	total	504	100
Race	Asian	9	1.79
	Black or African American	31	6.15
	White	351	69.64
	NA	113	22.42
	total	504	100
Ethnicity	Hispanic or Latino	8	1.59
	not Hispanic or Latino	319	63.29
	NA	177	35.12
	total	504	100

### 3.2 Screening and analysis of prognosis-related lncRNAs

**Figure 2:**
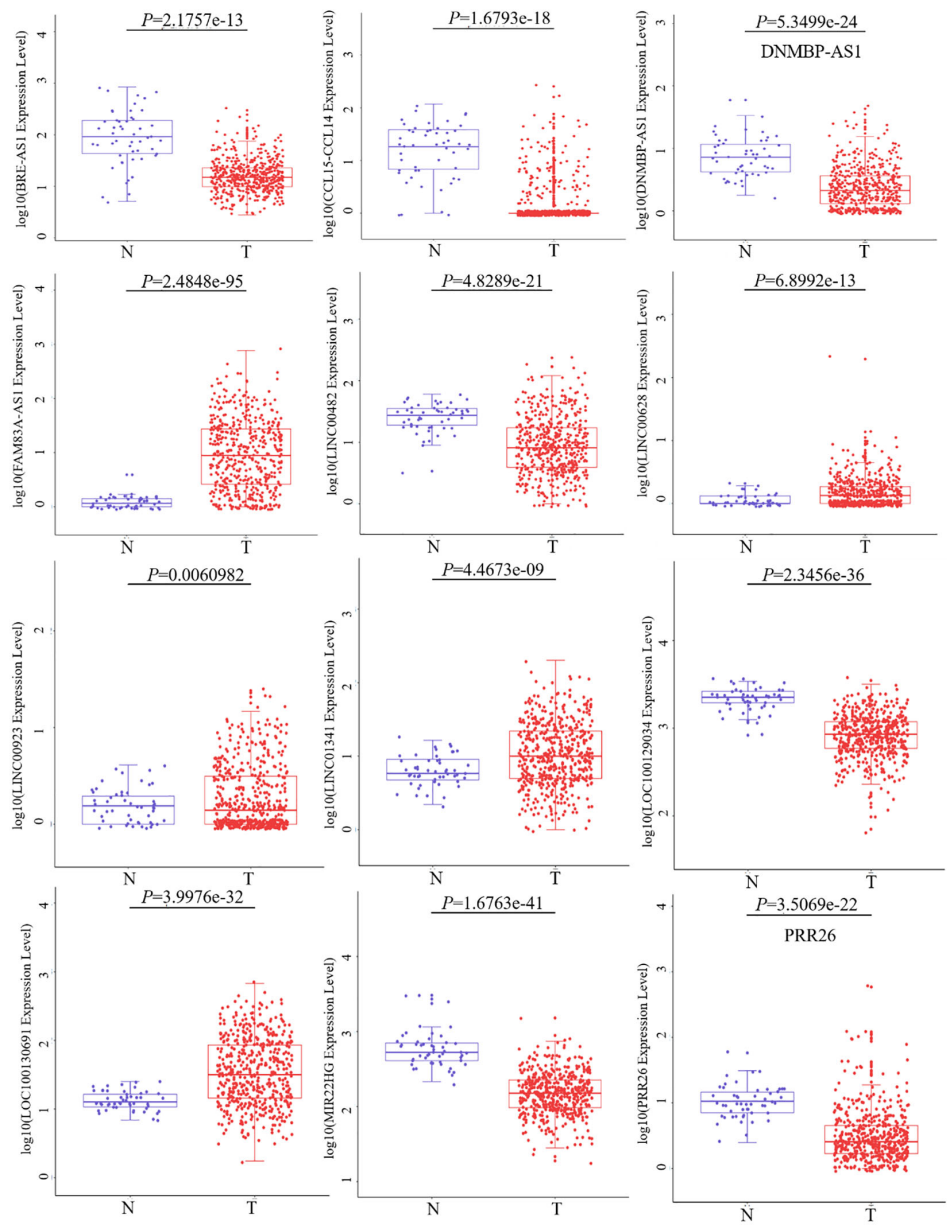
Differential expression of 12 lncRNAs between cancer and adjacent cancer samples.

By conducting an in-depth analysis of the correlation between overall survival (OS) and lncRNAs through univariate Cox proportional hazard regression, we successfully identified 271 lncRNAs with significant prognostic implications. Subsequently, a refined selection process involving multifactorial Cox proportional hazard regression analysis led to the identification of twelve lncRNAs. To provide insight into the expression patterns of these selected lncRNAs in LUSC and adjacent tissues, we performed t-tests and generated corresponding box plots. In the graphical representation, the designation “N” pertains to the adjacent cancer sample group, while “T” signifies the cancer sample group. Our analytical findings unveiled noteworthy disparities in the expression levels of these 12 lncRNAs between the cancer and adjacent cancer samples. Among the identified lncRNAs, seven (BRE-AS1, CCL15-CCL14, DNMBP-AS1, LINC00482, LOC100129034, MIR22HG, PRR26) exhibited significant downregulation, while five (FAM83A-AS1, LINC00628, LINC00923, LINC01341, LOC100130691) demonstrated notable upregulation, as depicted in **Figure 2**.

### 3.3 Prediction of target genes for lncRNA

**Figure 3:**
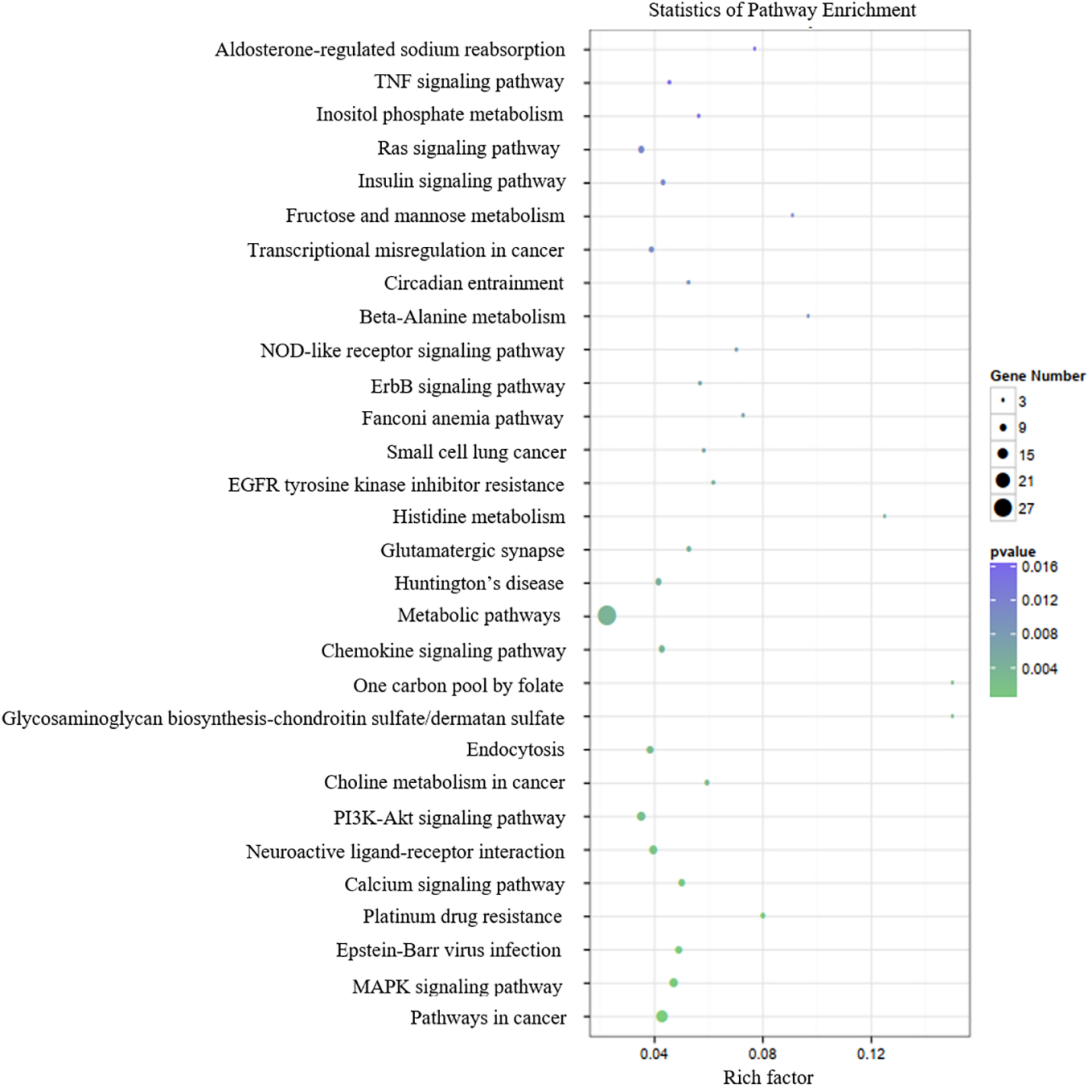
KEGG enrichment analyses of target genes of differentially downregulated lncRNAs.

**Figure 4:**
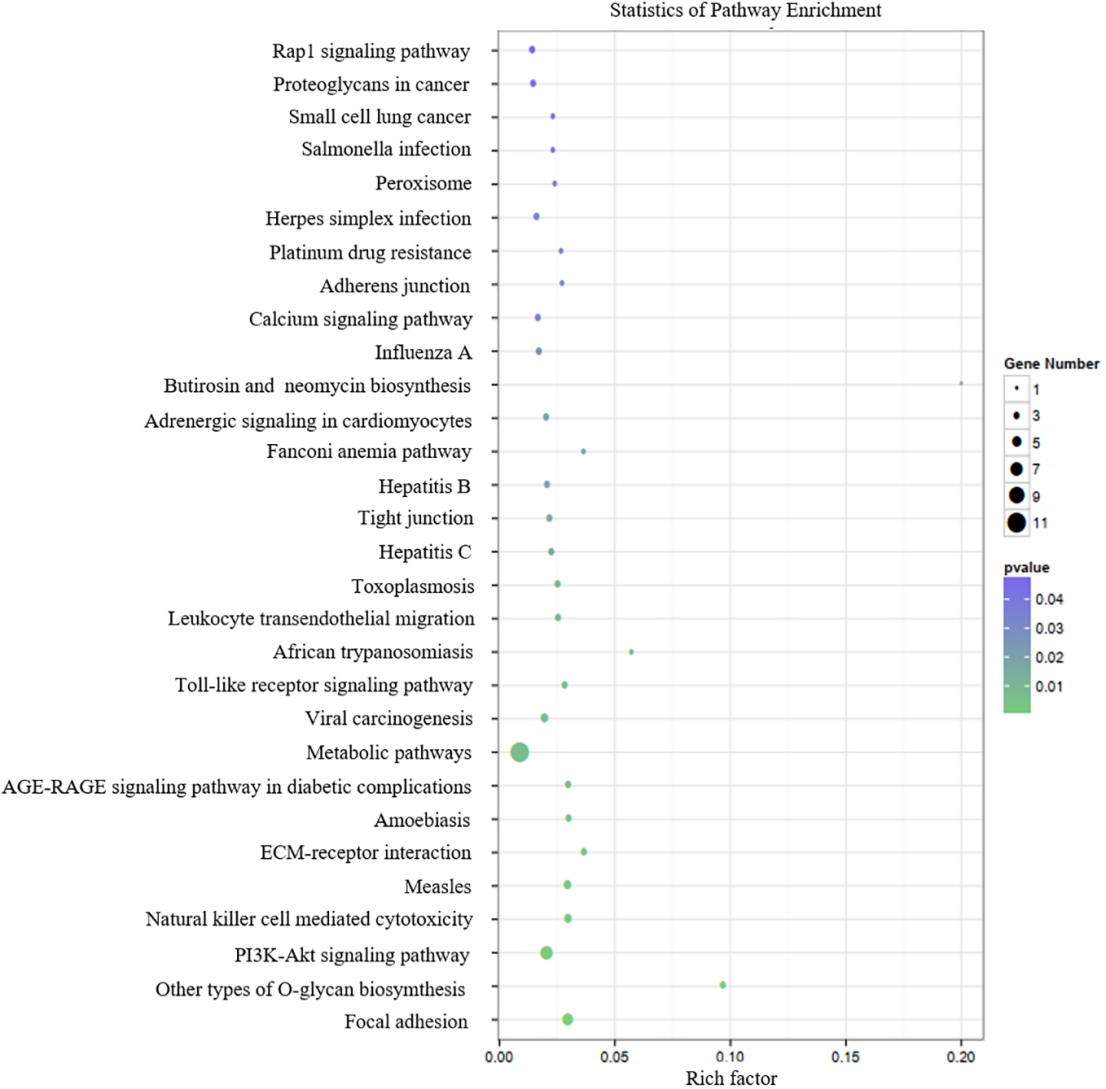
KEGG enrichment analyses of target genes of differentially upregulated lncRNAs.

**Figure 5:**
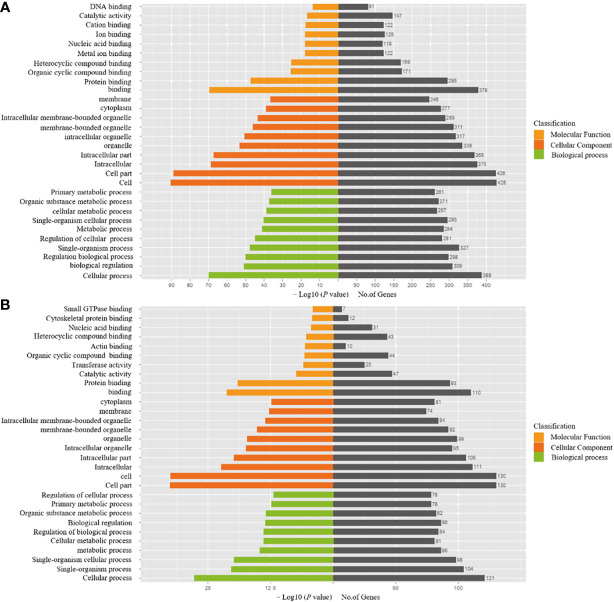
GO enrichment analyses of target genes of differentially expressed lncRNAs. **(A)** Functional analysis of differentially downregulated lncRNA target genes. **(B)** Functional analysis of differentially upregulated lncRNA target genes.

**Table 3** presents the predicted outcomes concerning the target genes associated with the 12 identified lncRNAs. It is noteworthy that the target genes of the differentially downregulated lncRNAs exhibited a pronounced enrichment in pivotal biological pathways, specifically those linked to metabolism, cancer, MAPK signaling, and PI3K-Akt signaling pathways (**Figure 3**). Conversely, the differentially upregulated lncRNAs demonstrated enriched target genes predominantly within metabolic and PI3K-Akt signaling pathways (**Figure 4**). Additionally, an overarching analysis of both downregulated and upregulated lncRNA target genes revealed a predominant enrichment in functions related to protein binding, cellular components, and cellular transformation, as depicted in **Figures 5A, B**. This extensive array of functions encompasses vital processes such as lung epithelial phosphorylation and cellular hypermetabolism, both of which play significant roles in the initiation and progression of LUSC.

**Table 3:** Statistical table of target gene prediction results of lncRNAs.

Gene	Cis num	Trans num
DNMBP-AS1	1	54
BRE-AS1	2	81
CCL15-CCL14	3	69
MIR22HG	2	228
LOC100129034	2	116
PRR26	0	0
LINC00482	2	0
LINC00923	0	0
LOC100130691	1	48
FAM83A-AS1	1	14
LINC00628	1	79
LINC01341	0	8

### 3.4 Verification of the proliferation ability of LINC00923 and LINC01341 in H226 cells

**Figure 6:**
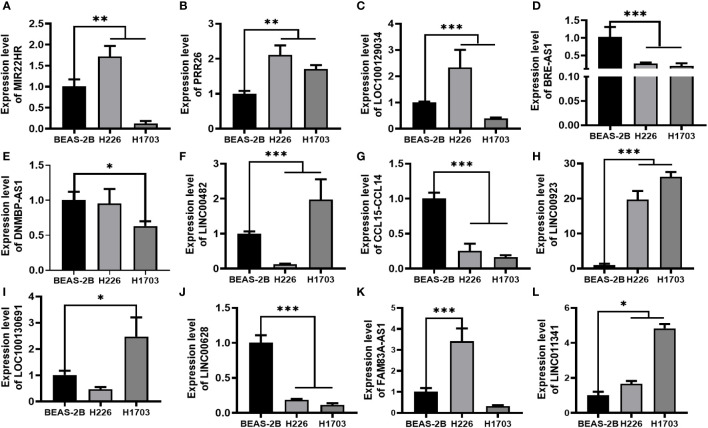
Verification of lncRNAs in H226 and H1703 cells. **(A–L)**. The expression levels of MIR22HG, PRR26, LOC100129034, BRE-AS1, DNMBP-AS1, LINC00482, CCL15-CCL14, LINC00923, LOC100130691, LINC00628, FAM83A-AS1, and LINC01341 in normal lung epithelial cells BEAS-2B and lung squamous cells H226 and H1703, respectively. *P<0.05; **P<0.01; ***P<0.001.

**Figure 7:**
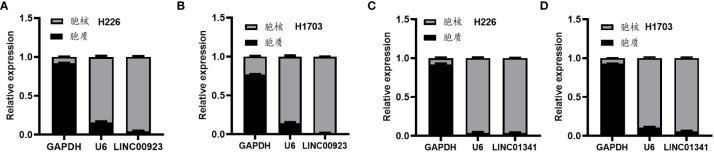
Illustration of the heightened expression of LINC00923 and LINC01341 within the nuclei of both H226 and H1703 cells. **(A, B)** The expression levels of LINC00923 in the nuclei of H226 and H1703 cells, respectively. **(C, D)** The expression levels of LINC01341 in the nuclei of H226 and H1703 cells, respectively.

**Figure 8:**
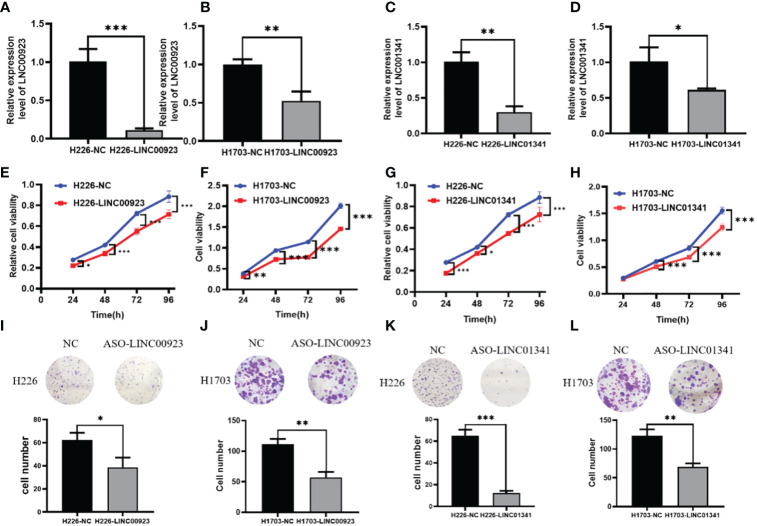
Illustration of the inhibitory effect of LINC00923 and LINC01341 knockdown on the proliferation of LUSC H226 and H1703 cells. **(A, B)** Evaluation of LINC00923 knockdown efficiency in H226 and H1703 cells. **(C, D)** Evaluation of LINC01341 knockdown efficiency in H226 and H1703 cells. **(E, F)** Assessment of proliferation capacity following LINC00923 knockdown in H226 and H1703 cells via CCK-8 assay. **(G, H)** Assessment of proliferation capacity following LINC01341 knockdown in H226 and H1703 cells via CCK-8 assay. **(I, J)** Determination of proliferation potential after LINC00923 knockdown in H226 and H1703 cells via colony formation assay. **(K, L)** Determination of proliferation potential after LINC01341 knockdown in H226 and H1703 cells via colony formation assay. *P<0.05; **P<0.01; ***P<0.001.

**Figure 9:**
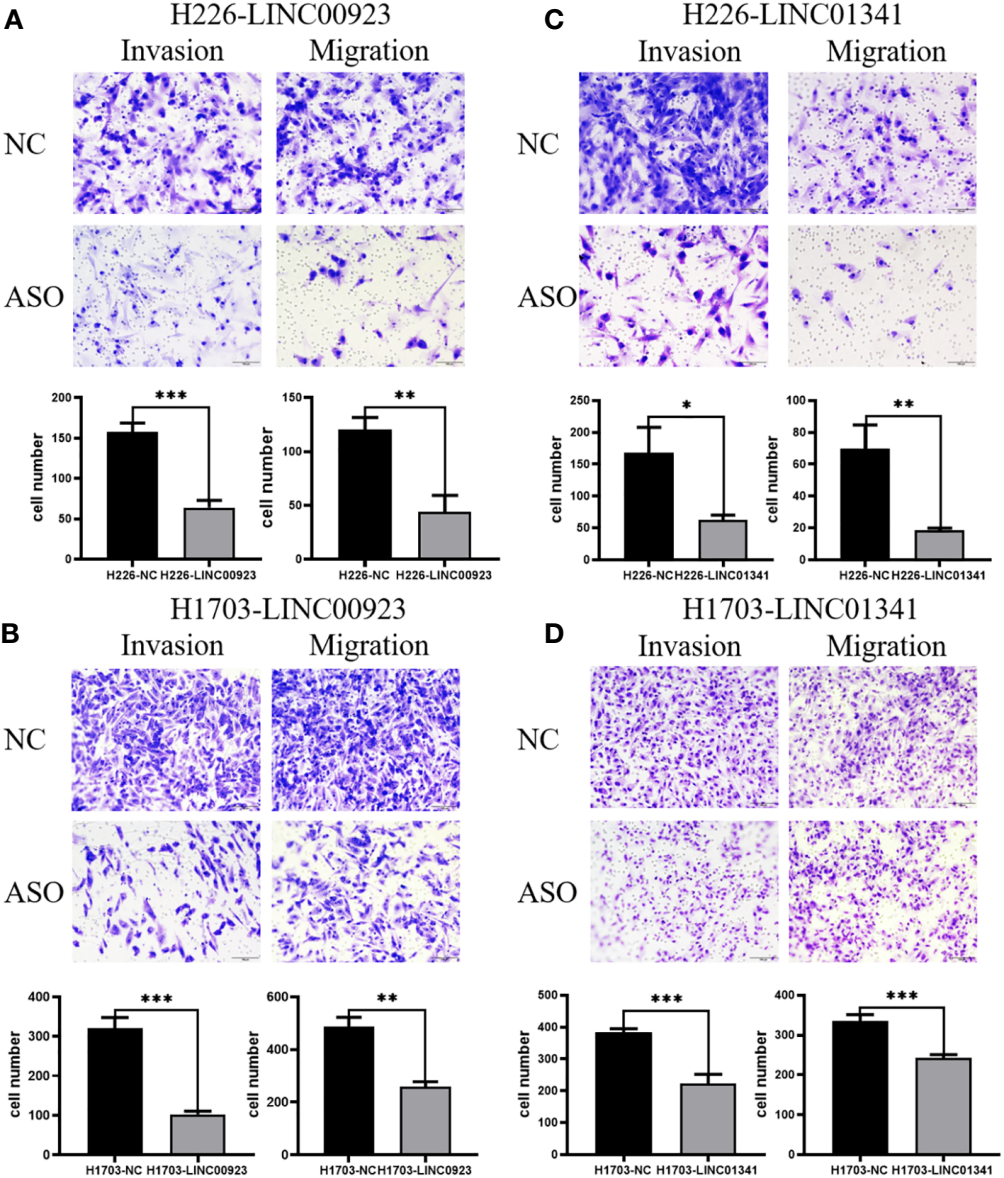
Illustration of the inhibitory effect of LINC00923 and LINC01341 knockdown on the invasion and migration of LUSC H226 and H1703 cells. **(A, B)** Evaluation of migration and invasion potential following LINC00923 knockdown in H226 and H1703 cells via transwell assay. **(C, D)** Assessment of migration and invasion potential following LINC01341 knockdown in H226 and H1703 cells via transwell assay. *P<0.05; **P<0.01; ***P<0.001.

In our assessment of the expression profiles of BRE-AS1, CCL15-CCL14, DNMBP-AS1, LINC00482, LOC100129034, MIR22HG, PRR26, FAM83A-AS1, LINC00628, LINC00923, LINC01341, and LOC100130691 within H226 and H1703 cells, we observed that relative to normal lung epithelial cells, BRE-AS1 and CCL15-CCL14 exhibited downregulation in both H226 and H1703 cells, while LINC00923 and LINC01341 exhibited upregulation in H226 and H1703 cells, respectively. These findings are graphically represented in **Figure 6**. The expression levels of the remaining lncRNAs in H226 and H1703 cells exhibited slight deviations from the predicted outcomes, which could be attributed to the inherent heterogeneity among tumor cells. Given the detectability of upregulated lncRNAs in patient fluids and various samples, their study vis-à-vis their impact on tumorigenesis is often more feasible. Consequently, LINC00923 and LINC01341 were prioritized for further investigation. Initially, we selected these upregulated lncRNAs, LINC00923 and LINC01341, to assess their nucleocytoplasmic expression in H226 and H1703 cells. Our analyses indicated that LINC00923 and LINC01341 were predominantly localized within the nuclei of H226 and H1703 cells, as depicted in **Figure 7**. Given the conspicuous nuclear presence of LINC00923 and LINC01341, we conducted a comprehensive evaluation of their roles in the proliferation, invasion and migration ability of LINC00923 and LINC01341 in LUSC H226 and H1703 cells. Employing Antisense Oligonucleotide (ASO) primers, we embarked on a series of experiments encompassing CCK-8 assays, colony formation assays, and transwell assays. These assays were conducted to assess the proliferation, invasion, and migration capabilities of H226 and H1703 cells in response to the knockdown of LINC00923 and LINC01341. The transfection efficiency was confirmed through quantitative reverse transcription PCR (qRT-PCR), as illustrated in **Figures 8A–D**. Our results from the CCK-8 assay (**Figures 8E–H**) and the colony formation assay (**Figures 8I–L**) demonstrated that the downregulation of LINC00923 and LINC01341 exerted inhibitory effects on the proliferation of H226 and H1703 cells. Furthermore, our transwell experiments corroborated that the suppression of LINC00923 and LINC01341 led to attenuated invasive and migratory capabilities of H226 and H1703 cells, as illustrated in **Figure 9**.

## 4 Discussion

LUSC is characterized by a notably unfavorable prognosis, with a 5-year survival rate of merely approximately 15% due to often delayed diagnoses (27–30). Conventional diagnostic techniques exhibit limitations in terms of sensitivity and specificity, which poses challenges for early detection. Consequently, there is a pressing need for novel biomarkers that can enhance molecular diagnostics and prognostic accuracy (31–38). While proteins have historically served as diagnostic biomarkers, lncRNAs present distinctive advantages, including their inherent stability, tissue-specific expression patterns, and amenability to detection in various physiological fluids (39–42). Numerous investigations have validated the utility of lncRNAs in effectively distinguishing patients with early-stage cancer from healthy controls, showcasing their capacity to provide valuable prognostic insights pertaining to metastatic potential and recurrence likelihood (43). For instance, in the realms of oesophageal cancer (44), colorectal cancer (45), lung adenocarcinoma (46), and pancreatic cancer (47), lncRNAs have emerged as independent markers with the potential to predict disease outcomes. Consequently, the burgeoning body of evidence underscores the prospective utility of lncRNAs as markers for LUSC.

Numerous carcinogenic lncRNAs, including PITPNA-AS1 (48), lncRNA ATB (49), and LINC00173. v1 (50), are pivotal regulators in LUSC (51). Our initial step encompassed the comprehensive screening of differentially expressed lncRNAs, from which we discerned 12 exhibiting significant prognostic relevance. Within this selection, seven lncRNAs (BRE-AS1, CCL15-CCL14, DNMBP-AS1, LINC00482, LOC100129034, MIR22HG, PRR26) exhibited downregulation, while five (FAM83A-AS1, LINC00628, LINC00923, LINC01341, LOC100130691) displayed upregulation. Notably, with the exception of lncRNA MIR22HG, lncRNA BRE-AS1, lncRNA FAM83A-AS1, and LINC00628, the differential expression of the remaining lncRNAs was previously undocumented in the context of LUSC, establishing their association with prognostic outcomes for the first time. Evidently, the silencing of lncRNA MIR22HG engenders the activation of cell survival/death signaling pathways, indicating the potential of lncRNA MIR22HG as a novel diagnostic and prognostic marker for LUSC (52). Furthermore, lncRNA BRE-AS1, through the upregulation of NR4A3, elicits inhibitory effects on the growth and survival of lung adenocarcinoma cells (53). Conversely, lncRNA FAM83A-AS1 exerts a promotional influence on A549 cell progression by elevating FAM83A expression, concurrently heightening HIF-1 levels within the lung adenocarcinoma α/glycolysis axis, thereby augmenting tumoral proliferation and migration (54, 55). Additionally, studies have illuminated the epigenetic interaction of LINC00628 with the LAMA3 promoter, culminating in the development of lung adenocarcinoma (56). Noteworthy differential expression of LINC00628 between lung adenocarcinoma and LUSC has been identified, further emphasizing its prognostic relevance (57). In summary, the lncRNAs delineated in this investigation, which possess significant prognostic implications, facilitate the initiation of lung adenocarcinoma via distinct mechanistic pathways while concurrently displaying shared attributes across both lung adenocarcinoma and LUSC. This observation provides a degree of assurance regarding the accuracy and reliability of the lncRNAs identified in our study.

This study indicates that the target genes governed by lncRNAs are predominantly enriched in pivotal biological processes, including metabolism, oncogenesis, MAPK signaling, and PI3K-Akt signaling pathways, while concurrently harboring functional attributes encompassing protein binding, cellular composition, and cellular transformation. These findings substantiate the prevailing literature concerning LUSC (58, 59). Notably, prior research endeavors have unveiled that LUSC exhibits differential protein expression profiles primarily characterized by enrichments in metabolic pathways and other signal transduction cascades (60). Moreover, our *in vitro* cellular experiments have successfully corroborated that two lncRNAs, which were computationally predicted to exhibit heightened expression levels in LUSC, indeed manifest augmented expression in LUSC cells. Conversely, two lncRNAs forecasted to exhibit diminished expression levels in LUSC cells did, indeed, demonstrate reduced expression. Furthermore, upon the knockdown of LINC00923 and LINC01341 in H226 and H1703 cells, the cellular attributes associated with proliferation, invasion, and migration were significantly attenuated. This empirical evidence, to a certain extent, substantiates the fidelity and reliability of our prediction results.

This study has been primarily constructed utilizing data sourced exclusively from the TCGA, thereby necessitating a noteworthy caveat regarding the lack of external dataset validation, thus constituting a limitation of our investigation. Future research endeavors can overcome this limitation by undertaking comprehensive multicohort analyses that amalgamate the prognostic value of the identified lncRNAs with available expression datasets encompassing LUSC. Moreover, the current study lays a foundation for potential cellular investigations aimed at elucidating the mechanistic links between LINC00923 and LINC01341 and their putative target genes within the metabolic and PI3K-Akt signaling pathways. Such investigations would furnish additional empirical substantiation, thereby bolstering the candidacy of LINC00923 and LINC01341 as viable biomarkers for prognosticating LUSC. In summary, this study has successfully winnowed down a compendium of 12 lncRNAs exhibiting prognostic relevance. Subsequent cellular validation endeavors have identified LINC00923 and LINC01341 as prospective biomarkers with the potential to serve as predictive indicators for LUSC.

## Data availability statement

The datasets presented in this study can be found in online repositories. The names of the repository/repositories and accession number(s) can be found below: https://www.ncbi.nlm.nih.gov/, https://www.jianguoyun.com/c/sd/17d0c4c/4f4303a7e2ec9502.

## Author contributions

Conception and design: YC. Acquisition of data: YW and YC. Analysis and interpretation of data: YC, MZ, YZ, XS, and WK. Writing and review of the manuscript: YC and XZ. Revision of the manuscript and study supervision: GS. All authors contributed to the article and approved the submitted version.
